# Smoking Ban Law in Chile: Impact in Newborns’ Birth Weight by Women’s Age Groups and by City Population Density

**DOI:** 10.3389/ijph.2022.1605087

**Published:** 2022-12-12

**Authors:** Giovanna Valentino, Ana Ortigoza, Lorena Rodriguez Osiac, Tamara Doberti, Pricila Mullachery, Carolina Nazzal

**Affiliations:** ^1^ Carrera de Nutrición, Departamento de Ciencias de la Salud, Facultad de Medicina, Pontificia Universidad Católica de Chile, Santiago, Chile; ^2^ School of Public Health, University of Chile, Santiago, Chile; ^3^ Urban Health Collaborative, Dornsife School of Public Health, Drexel University, Philadelphia, PA, United States; ^4^ Department of Health Services Administration and Policy, College of Public Health, Temple University, Philadelphia, PA, United States

**Keywords:** tobacco, maternal & child health, Latin America, smoke-free policy, low birth weight, Chile

## Abstract

**Objectives:** We examined the short-term impact of the Smoking Ban Law (SBL) enacted in Chile in 2013 on low birth weight (LBW) rates in cities and its differential effects by different maternal age groups and city density.

**Methods:** We included 885,880 live births from 21 Chilean cities of ≥100,000 inhabitants. We examined the smoking and LBW prevalence distribution before and after the SBL. Through Poisson mixed effect models, we determined whether a meaningful change in LBW rate occurred after SBL implementation in the whole sample and stratified by city population density and maternal age group.

**Results:** LBW prevalence remained stable before and after the SBL implementation (6.1% and 6.3%, respectively), while women’s smoking prevalence had a relative reduction of 25.9% (*p* < 0.00001). No significant changes in LBW rate occurred after the implementation of SBL in the total sample or stratified by city density tertiles or maternal age groups.

**Conclusion:** SBL implementation did not show short-term impact on LBW rate in Chile. Further studies need to examine long-term impact of SBL on low birthweight.

## Introduction

More than 20 million newborns with low birth weight (LBW, defined as birthweight <2,500 gr) occur worldwide each year, which is associated with detrimental effects on infant development and chronic illness during adulthood [1]. The prevalence of LBW in the population is made up of term newborns who, because of fetal growth retardation, are small for their gestational age (SGA) and pre-term newborns, whose birth weight might be adequate for their gestational age or are also SGA. Maternal smoking and exposure to second-hand smoking during pregnancy have been associated with fetal growth retardation, reducing mean birthweight and increasing the risk of LBW in the offspring [2–6].

In Chile, the tobacco burden is the highest among Latin America with a current smoking prevalence of 33% [7]; however, the smoking prevalence among women is currently lower compared to previous years. Data from National Health Survey showed that in 2003 smoking prevalence among women of all ages was 38,3%, while this prevalence was significantly lower at 29,1% in 2016–2017 [8, 9]. Smoking prevalence was higher among the population aged 20–29 years old but experienced a steep decrease between 2003 and 2016–2017 (from 60.5% to 41.1%) [9]. Part of the decrease in smoking prevalence particularly among the young population, and women, could be ascribed to the implementation of strong and progressively tobacco smoking banning nationwide. In 1995 the tobacco law was enacted (Law No. 19419), which restricted the consumption, sale, and advertising of products made with tobacco in Chile [10]. In March 2013, a new law (No. 20660) increased these restrictions by prohibiting smoking in closed places accessible to the public or for collective commercial use, on terraces that are not outdoors or have a roof that is attached to a wall and in sports venues, stadiums, or gyms. Additionally, this law forbade the sale of cigarettes within a radius of 100 m around schools, the sale or free delivery of cigarettes in units or loose, and in packages containing quantities of less than 10 units, all kinds of tobacco advertising, smoking in programs and advertising on TV shows broadcasted during minors’ hours. This law was enforced almost immediately and simultaneously across the nation, and it also regulated tobacco labeling [11].

Similar smoking ban law (SBL) implementations in other parts of the world (United States, China, Australia, United Kingdom) have shown to have a greater impact in reducing smoking among young people, possibly due to a delay in smoking initiation habits at early ages [12–15].

Other studies have shown that the effectiveness of SBL in reducing tobacco behavior in the population could be modified by urban environments. With a higher number of public places for social interaction, more dense cities have experienced a greater reduction in second-hand smoking than less dense cities [16, 17]. Some studies, the majority in high-income countries, have assessed the impact of SBLs on reducing LBW over the years. However, no study has examined variations in the impact of SBLs among women in different age groups or across cities with different population density [18–20].

In Latin America and the Caribbean (LAC), smoking policies have been implemented in 23 of 35 countries [21]. There are still countries that do not have regulation for smoking in public places and these have a higher prevalence of LBW in the population [21]. Knowing whether SBL contribute to reducing LBW prevalence in the short run could support the continuation of smoking restrictions in Chile, and the promotion of SBL implementation in other countries from LAC and the Global South that have not yet implemented public smoking restrictions.

This study aimed to examine whether implementation of SBL had an impact on the LBW rate and whether this effect is different among women of different age groups and cities with different population density. We hypothesized that SBL implementation contributed to reducing LBW prevalence, and that this effect is more significant among younger women and in more dense cities.

## Methods

### Study Setting and Data Sources

We performed an ecological analysis using data from SALURBAL study, which has compiled data for live births, population, and health from 371 cities with ≥100,000 inhabitants across 11 Latin-American countries (Argentina, Brazil, Chile, Colombia, Costa Rica, El Salvador, Guatemala, Mexico, Nicaragua, Panama, and Peru) [22]. Each city was defined geographically by administrative units (municipios) that encompassed the urban extent of the city in 2010 using satellite imagery [22]. For this analysis we used individual data from live birth registries linked to cities by the maternal place of residence and population projection from census for each city in Chile between 2011 and 2015 (*n* = 21 cities). We also used Census and National Health Survey databases for collection of sociodemographic and smoking prevalence, which data collection methods has been described before [22].

#### Intervention/Exposure

SBL was implemented almost immediately and simultaneously across the nation in March 2013. We then defined exposure as at least a complete month of having received the intervention. Following this rationale, the pre-intervention period was considered from January 2011 to March 2013 and the post-intervention period from April 2013 to December 2015.

#### Sample

**FIGURE 1:**
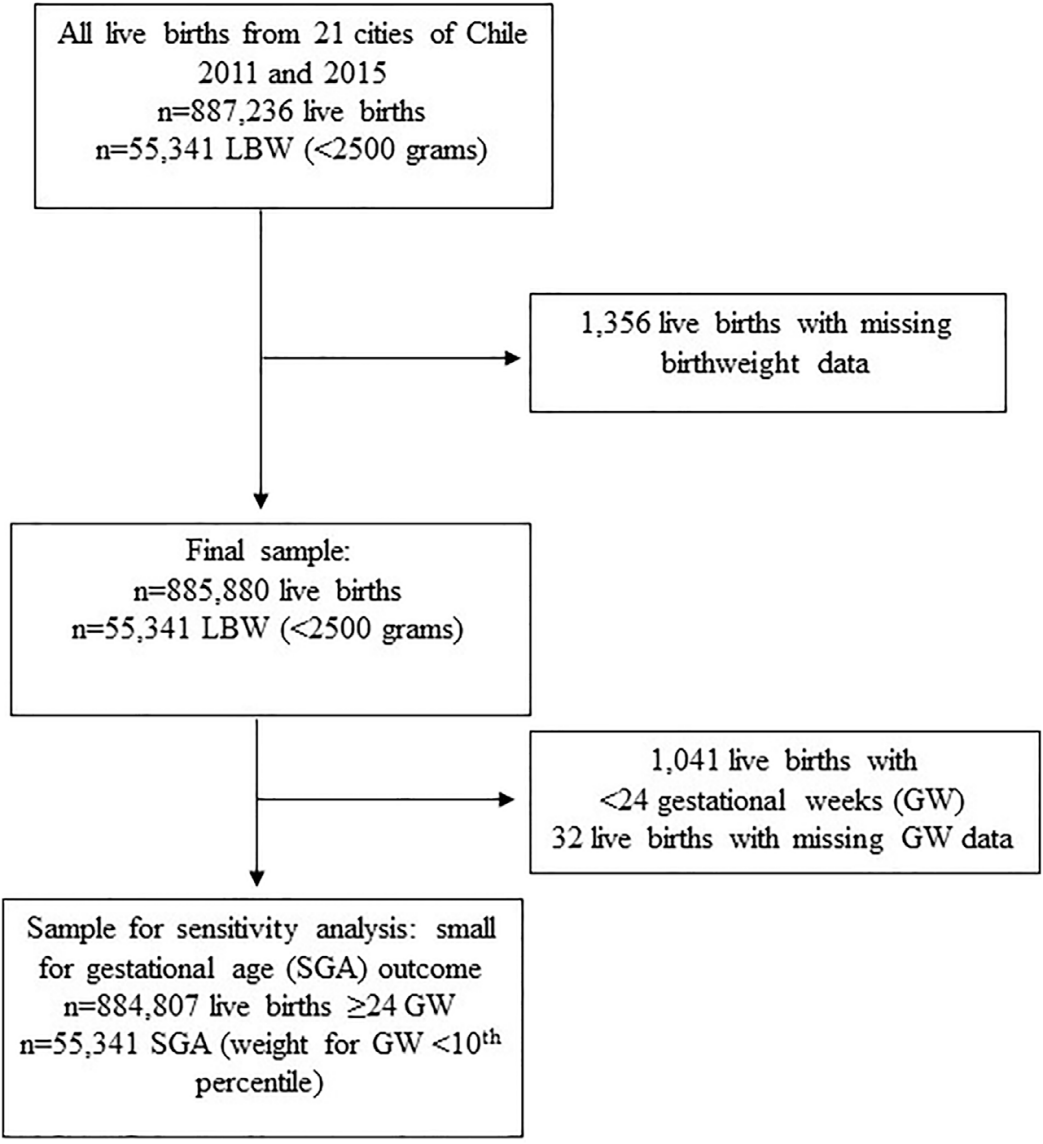
Sample selection flowchart. LBW: Low birth weight (birthweight <2,500 g), Chile, 2011–2015.

We included all live births that occurred during the two above mentioned periods in all cities with ≥100,000 inhabitants (*n* = 21) from Chile. Figure 1 shows the sample selection flowchart.

#### Outcome

LBW rate was defined as the number of newborns with birthweight less than 2.5 kg per 1,000 live births in each city per trimester during January 2011- March 2013 (pre-intervention period, 9 trimesters) and during April 2013–December 2015 (post-intervention period, 11 trimesters).

#### Other Variables

We included in our analysis variables that are shown to be related with the prevalence of LBW and with smoking behavior in the population.

Maternal age groups: categorized as <20 years old, 20–34 years old, and ≥35 years old.

Population density: To determine city population density, we calculated the mean city density for the period between 2011 and 2015 for each city, which was calculated as the number of people living per km^2^ of built-up area within the geographic boundaries of the municipios [22]. Distribution of cities countrywide by population density categories is shown in Supplementary Figure S1.

Smoking prevalence: we determined the prevalence of current smokers in cities by sex and age groups using National Health Survey databases (2009–2010 and 2016–2017). Current smokers were defined as those who smoked daily (≥1 cigarette/day) or occasionally (<1 cigarette/day) in the past 6 months.

### Statistical Analysis

We described trends in LBW and smoking prevalence among women, by tertiles of city population density, and maternal age groups over the pre- and post-intervention period. We used a mixed effects analysis of variance (ANOVA) model to compare changes on smoking prevalence before and after the SBL implementation (time effect within cities) and differences between tertiles of population density and maternal age categories. Regression discontinuity was used to determine whether a change in the prevalence of LBW occurred immediately after Chile passed the SBL. Regression discontinuity analysis allows the assessment of the average treatment effect of an intervention by comparing observations or trends closely before and after the implementation of such intervention [23]. The selection of a short time-frame was used to reduce possible bias due to the effect of other interventions or other time-variant exposures on LBW rate.

We examined whether changes in LBW rate were associated with SBL intervention through Poisson mixed effect models. We accounted for city clustering by including a random intercept for cities. The time unit was defined as calendar trimesters as shown in the formula below.

$$\mathrm{Ln}{\left(Y\right)}_{ti}=\beta_{0i}+\beta_{1}t_{i}+\beta_{2}X_{ti}+\beta_{3}X_{ti}*ti$$

$$Offset=\ln E_{ti}$$

$$b_{oi}=\gamma_{oo}+U_{oj}$$

*Yti* denotes the rate of LBW in trimester t and city i. We considered *t* as the number of trimesters, where t = 0 corresponds to the first trimester of the whole period. The period before the implementation of the smoking ban corresponds to 9 trimesters with *t* ≥0 and ≤8, while the ‘post-intervention’ corresponds to 11 trimesters with t > 8. X is a dummy variable that takes the value 0 in the pre-intervention period and 1 in the post-intervention period. Our parameters of interest were:

• β_1_ captures the change in LBW associated with a time unit increase (i.e., change in slope) in the period before the SBL (pre-intervention trend).
• β_2_ captures the level change in the LBW immediately after the SBL becomes effective, which ultimately corresponds to the “gap” between the pre and post intervention trends.
• β_3_ as it indicates the change in the temporal trend (i.e., change in slope) after the intervention.

If the smoking ban legislation has an immediate reduction in the rate of LBW, the coefficient β_2_ should be a negative value and significantly different from zero. If SBL has, on the opposite, a gradual influence in the reduction of LBW rate we will find that β_3_ coefficient will be of negative value and significantly different from zero.

For assessing differential effects by maternal age and city density, we stratified the main analysis by maternal age groups and by tertiles of city population density. We also performed a second model adjusted by seasonality using quarters of each year as dummy variables (1 = January–March, 2 = April–June, 3 = July–September, 4 = October–December).

We performed multiple sensitivity analyses. Firstly, to account for a possible delay in the effect of the policy due to the inclusion of live births that may have been exposed to “pre – intervention” smoking law conditions for some part of their gestation, we added lags at 1, 2, and 3 calendar trimesters (3, 6, and 9 months) after the intervention using the main analytical approach (trimesters cut-off points: July 2013, October 2013, January 2014, respectively). Secondly, we used a more refined time analysis where we calculated and modeled monthly rates of LBW (instead of calendar trimesters) from January 2012 to December 2013 to estimate the same coefficients as in the main model. Third, we analyzed LBW rate as a continuous outcome using mixed effects models. As total live births varied across cities affecting the LBW rate, we considered weights in the models (weight = √number of live births) for each city. Lastly, we carried out the main analytical strategy only including LBW infants who had a diagnosis of small for gestational age (SGA) among newborns with ≥24 GW. We defined SGA as those with birthweight lower than the 10^1h^ percentile according to Alarcón- Pittaluga curves built for Chilean newborns older than 24 GW [24].

## Results

Table 1 shows baseline and relative changes in live birth, maternal, and city characteristics by tertiles of population density for the pre and post intervention periods for all 21 cities. Overall, baseline LBW prevalence was 6.1%. Before SBL implementation, cities with medium- density (Tertile 2 = 6,198–8,132 persons/km^2^) had a lower LBW prevalence compared to those with the lowest and highest density (Tertiles 1 and 3). Overall baseline prevalence of preterm newborns (<37 GW) was 7.8% and high- density cities (Tertile 3 ≥8,134 persons/km^2^) had a higher rate of preterm births compared to those with lower density. LBW prevalence remained quite stable over time, with a slight increase during the post-intervention period at the expense of pre-term newborns and in cities with medium density (Tertile 2, Table 1).

**TABLE 1:** Maternal and live birth characteristics before and after the implementation of the smoking ban law by tertiles of city density in law in all 21 cities of 100K+ residents in Chile, 2011–2015.

	Births before smoking ban law (January 2011-March 2013)				Relative change between before and after the implementation of SBL			
	Total (*n* = 398,101)	Tertile 1 (*n* = 44,482)	Tertile 2 (*n* = 76,752)	Tertile 3 (*n* = 276,867)	Total (*n* = 487,779)	Tertile 1(*n* = 54,844)	Tertile 2 (*n* = 93,175)	Tertile 3 (*n* = 339,760)
Live birth characteristics								
Total LBW, n (%)	24,447 (6.1%)	2,801 (6.3%)	2,624 (5.7%)	17,293 (6.3%)	3.3%	3.2%	5.3%	1.6%
LBW ≥37 GW, n(%)	6,493 (1.8%)	862 (2.1%)	1,151 (1.6%)	4,480 (1.8%)	0%	4.8%	0%	0%
<37 GW, n(%)	31,066 (7.8%)	3,159 (7.1%)	5,529 (7.2%)	22378 (8.1%)	3.8%	5.6%	8.3%	2.5%
Maternal characteristics								
<19 years old, n(%)	54,135 (14%)	6,614 (15%)	11,091 (14%)	36,430 (13%)	−21.40%	−20.0%	−14.3%	−15.4%
20–34 years old, n(%)	275,730 (69%)	30,947 (70%)	53,062 (69%)	191,721 (69%)	2.9%	1.4%	2.9%	2.9%
≥35 years old, n(%)	68,236 (17%)	30,947 (16%)	12,599 (16%)	48,716 (18%)	5.9%	6.3%	6.3%	0.0%
Maternal education ≥12 years, n(%)	302,961 (76%)	32,616 (73%)	56,840 (74%)	213,505 (77%)	5.3%	5.5%	5.4%	3.9%
City characteristics								
City density, inhabitants/km^2^	7,238 (1805)	5,605 (357)	6,704 (674)	9,405 (1,164)	3.2%	3.1%	3%	3%
City population (inhabitants), p50 (IQR)	216,138 (188,751)	154,913 (129,441)	213,212 (253,701)	348,604 (722,791)	3.5%	3.2%	3%	4%
Women at reproductive age, % (min-max)	52.6% (48.1–55.5)	52.0% (48.1–53.7)	52.8% (50.2–55.5)	53.1 (50.6–54.6)	−1.3%	−1.5%	−1.5%	−1.1%
General fertility rate, (min-max)	56.9 (49.5–71.6)	58.3 (50.9–69.3)	54.5 (49.5–59.7)	57.8 (49.5–71.6)	−2.5%	−1.0%	−3.1%	−3.1%
Overall Smoking prevalence, mean (min-max)	39.3% (33.9–46.3)	38.8% (35.2–42)	40.2% (38.5–45.4)	38.9% (33.9–46.3)	−18.8%^a^	−13.7%^a^	−21.1%^a^	−21.6%^a^
Smoking prevalence in women, mean (min-max)	37.8% (30.6–44.7)	37.6% (30.6–43.1)	38.6% (33.6–44.7)	37.1% (31.9–40.9)	−25.9%^a^	−17.6%^a^	−32.1%^a^ ^,^ ^b^	−27.8%^a^
W 20–34 years old	45.8% (38.7–53.3)	46.2% (39.5–53.3)	47.0% (41.4–52.7)	44.1% (38.7–50.9)	−30.1%^a^	−24.5%^a^	−35.5%^a^ ^,^ ^b^	−30.4%^a^
W 35–49 years old	39.6% (32.8–47.0)	40.0% (33.6–47.0)	40.7% (35.3–46.3)	38.0% (32.8–44.5)	−26.5%^a^	−20.5%^a^	−32.4%^a^ ^,^ ^b^	−26.6%^a^

^a^ Indicates a significant change after the SBL implementation (*p* < 0.05).

^b^ Indicates significant difference with tertile 1 (*p* < 0.05). Relative change was calculated as the difference between mean, p50 or prevalence (categorical variables) before and after the SBL (Relative change = post-pre)/pre).

SBL, smoking ban law; LBW, Low birth weight (birthweight <2,500 g); GW, gestational weeks at birth, Women at reproductive age: proportion of women between 15 and 49 years old/total of women population; General fertility rate: number of live births per 1,000 women at reproductive age (15–49 years old). Categoric variables are expressed in frequency (n) and prevalence (%). Continuous variables are expressed in average and standard deviation or median (p50) and interquartile range (IQR) when indicated.

Before the implementation of SBL, overall smoking prevalence was similar across city density tertiles (37.1%–38.6%). Women aged 20–34 years had a higher smoking prevalence compared to those ≥35 years old at all tertiles for population density (*p* < 0.0001). Smoking prevalence had a relative decrease of 18.8% in the overall population after SBL with a higher decrease in women compared to men (−25.9% vs. −12.2%, respectively; *p* = 0.04). Mid-density cities had a higher decrease compared to lower-density cities (−32.1% vs. −17.6%, respectively; *p* = 0.01), whereas women aged 20–34 years old had a higher decrease of smoking prevalence compared to those ≥35 years (−30.1% vs. −26.5%; *p* = 0.08), although differences were not statistically significant.

**FIGURE 2:**
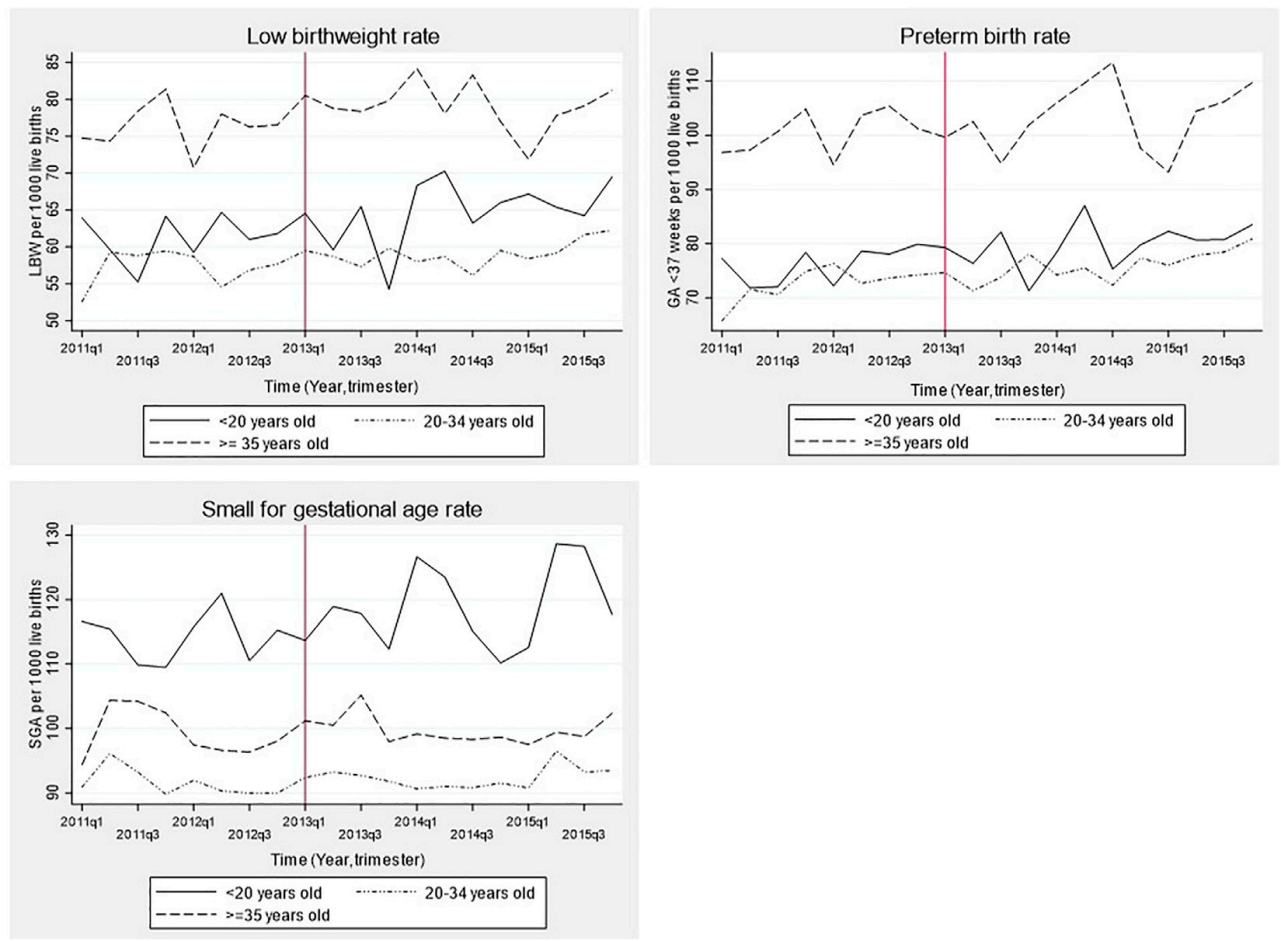
Time series of birth outcomes before and after the implementation of the smoking ban law in Chile stratified by maternal age group, 2011–2015. LBW: Low birth weight (birthweight <2,500 g), GA: Gestational age, SGA: Small for gestational age. Time was a discrete variable measured as 20 trimesters or quarters: from the first trimester of 2011 (time = 0 = 2011q1) to the last trimester of 2015 (time = 19 = 2015q4). The red line shows the implementation of the smoking ban law (trimester including March 2013 = 2013q1).

Figure 2 shows time series for LBW, SGA and preterm rate stratified by maternal age categories. LBW and preterm birth rate before and after SBL implementation was highest among women ≥35 years old (7.8% and 10.3%, respectively; *p* < 0.0001 for comparison with other age categories) and lowest among those between 20 and 34 years old (5.8% and 7.5%, respectively). Prevalence of SGA was highest among women aged <20 years old and lowest among those with 20–34 years (11.7% vs. 9.2%, respectively; *p* < 0.0001).

**FIGURE 3:**
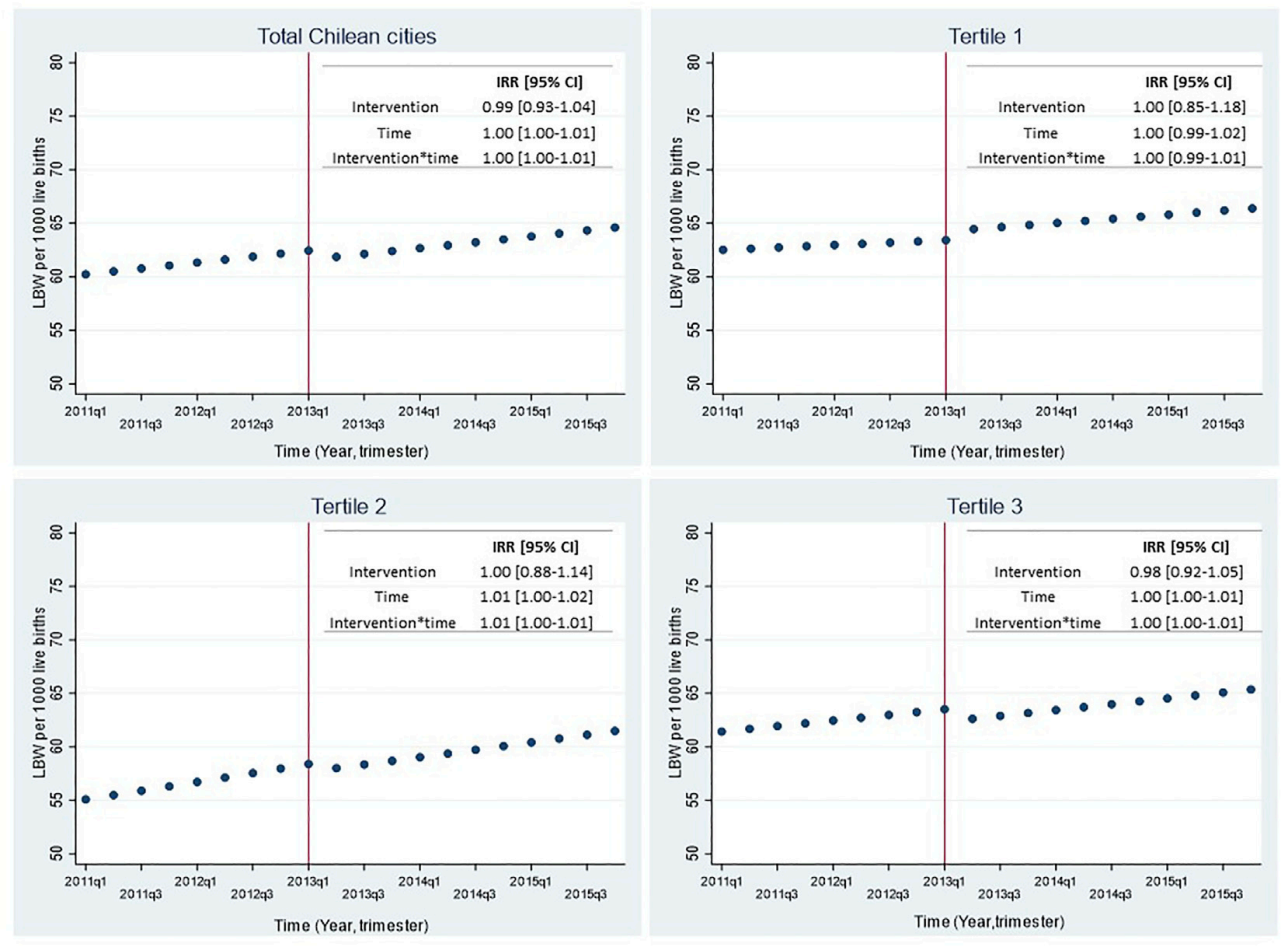
Effect of smoking ban law implementation over low birthweight rate in all 21 Chilean cities and by tertiles of city density, 2011–2015. LBW: Low birth weight (birthweight <2,500 g); IRR: Incidence Rate Ratio; 95% CI: 95% Confidence Interval. The top left figure shows the predicted LBW rate for all 21 Chilean cities; the top right figure shows the predicted LBW rate for cities of the lowest tertile of population density (≤6,153 pers/km^2^); the bottom left figure shows the predicted LBW rate for cities of the intermediate tertile of population density (6,198–8,132 persons/km^2^); the bottom right figure shows the predicted LBW rate for cities of the highest tertile of population density (≥8,134 persons/km^2^). Time was a discrete variable measured as 20 trimesters or quarters: from the first trimester of 2011 (time = 0 = 2011q1) to the last trimester of 2015 (time = 19 = 2015q4). The red line shows the implementation of the smoking ban law (trimester including March 2013 = 2013q1).

**FIGURE 4:**
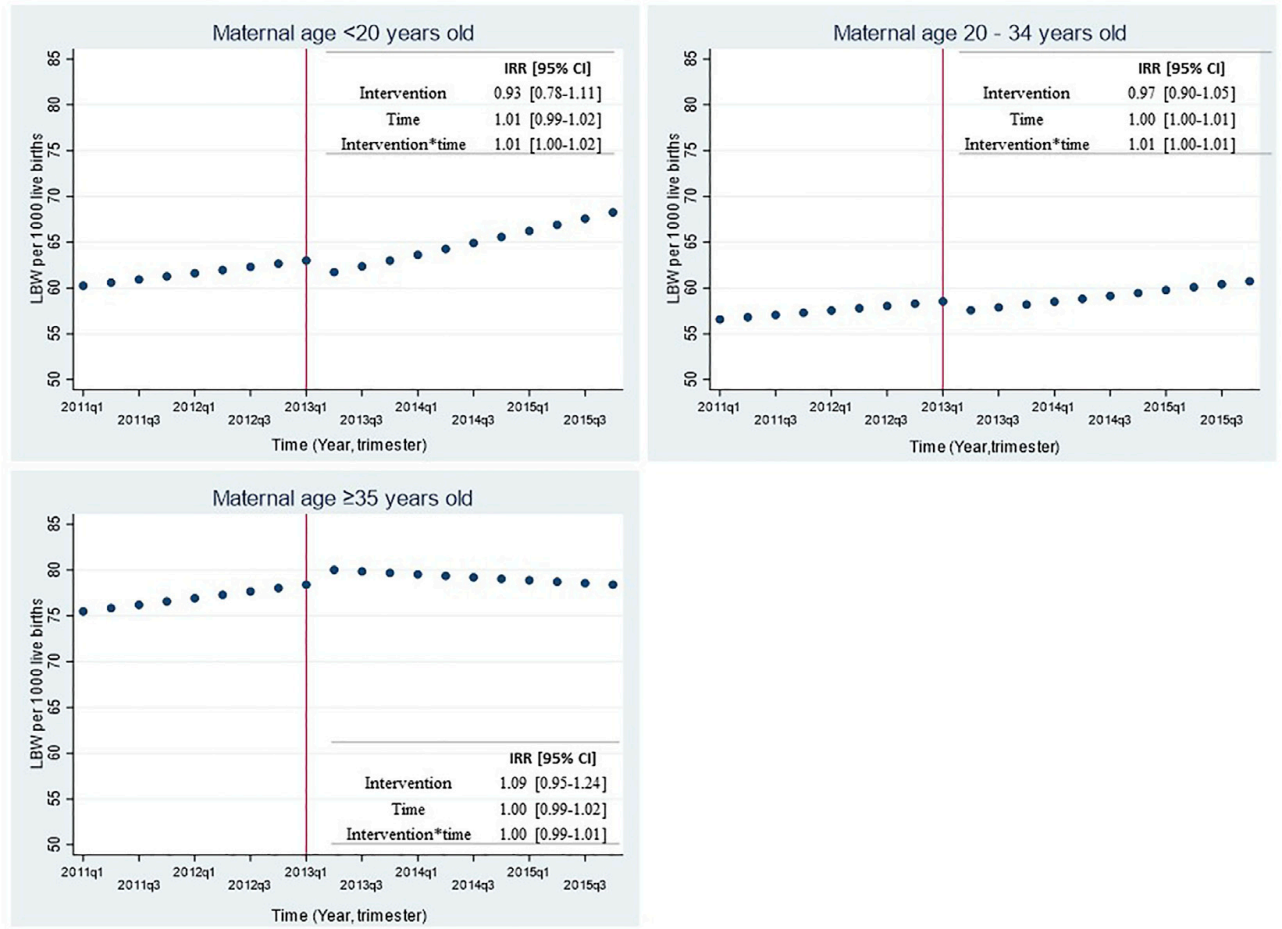
Effect of smoking ban law implementation over low birthweight rate in all 21 Chilean cities by maternal age group 2011-2015. LBW: Low birth weight (birthweight <2500 grams); IRR: Incidence Rate Ratio; 95% CI: 95% Confidence Interval. The top left figure shows the predicted LBW rate for mothers younger than 20 years old; the top right figure shows the predicted LBW rate for mothers between 20 and 34 years old; the bottom left figure shows the predicted LBW rate for mothers with 35 years or older. Time was a discrete variable measured as 20 quarters: from the first quarter or trimester of 2011 (time=0=2011q1) to the last quarter or trimester of 2015 (time=19=2015q4). The red line shows the implementation of the smoking ban law (March 2013).

Figure 3 shows trends in LBW rate before and after SBL implementation for the whole sample (21 cities) and stratified by city density tertiles with their respective incidence rate ratios (IRR) and 95% confidence interval. No significant changes were observed in LBW rate immediately after the intervention (IRR = 0.99 [0.93–1.04]; *p* = 0.66). Similar results were observed when stratifying by city density tertiles (Figure 3), by maternal age (Figure 4) or when adjusting the models by seasonality (Supplementary Table S1).

In the sensitivity analysis, similar results were observed when using months as time-unit (IRR = 0.99 [0.91–1.08]; *p* = 0.87) and when accounting for lag effects of 3, 6, and 9 months (IRR = 0.98 [0.92–1.04], *p* = 0.50; IRR = 0.98 [0.90–1.05], *p* = 0.53; IRR = 0.96 [0.88–1.06], *p* = 0.44, respectively; Supplementary Figure S2). Sensitivity analysis using LBW rates made of small for gestational age (SGA) as the outcome, did not showed significant changes after the SBL implementation (IRR = 0.98 [0.94–1.02], *p* = 0.49); similar null results were found when using linear mixed effect models with LBW rate as a continuous outcome (Supplementary Table S1).

## Discussion

This study examined changes in smoking and LBW prevalence previous and after the implementation of SBL in Chile and assessed whether these changes varied by city population density and maternal age. Although no significant changes in LBW prevalence have been found immediately after the implementation of SBL in the overall sample of cities and by city density and maternal age, we described significant lower smoking prevalence after SBL implementation in the overall population and across all tertiles of city density, particularly among those in mid-density cities and among women aged 20–34 years old after the implementation of SBL.

We have found no meaningful change in LBW rate after the implementation of SBL. Results from regional or country-level studies using a similar approach are mixed. On one hand, a German study assessing the impact of SBL on perinatal outcomes on Bavaria, showed no significant immediate effect on most of the perinatal outcomes under study (LBW, preterm birth, SGA, and still birth) except from extreme pre-term births [25]. On the other hand, a study in Quebec examining the immediate effects of the SBL and after 3,6, and 9 months on perinatal outcomes showed a significant reduction in SGA, preterm birth and LBW rate only when considering a 9-month lag period [26]. They identified several factors that may explain the heterogeneous findings across studies assessing the effect of smoke-free legislation on pregnancy outcomes, namely: 1) different policy environments in terms of smoking prevalence and smoking norms, 2) the presence of existing legislation prior to the smoke-free legislation under investigation, and 3) differences in policy implementation and enforcement [26]. Peelen et al., who investigated whether immediate changes in perinatal outcomes occurred following SBL and the introduction of key tobacco control policies (taxes and mass media campaign) in the Netherlands reported a reduction in SGA births, but not in LBW [27]. Unlike to those results, we did not find an effect of SBL when considering 3, 6, 9 months- lag periods or SGA rate as an outcome on the sensitivity analysis.

In the case of Chile, the null results of our study could be explained in part by the fact Chile had a long trajectory of public health promotion and programs to reduce tobacco consumption and exposure during critical physiological states such as pregnancy and childhood. These strategies might increase health-conscious behavior among pregnant women, who, prior to the implementation of the smoke-free legislation, already avoided smoke and exposure to secondhand smoke.

Additionally, LBW rates in Chile are low. In 2015, national LBW rate was 6.2% (below the world rate of 14.6%) [28]. We observed lowest rates in mid-density cities (population density between 6198 & 8132 persons/km^2^ Tertile 2), and, among mothers aged 20–34 years. Chile has implemented interventions to improve perinatal and infant health prior to anti-smoking legislation, such as the creation of the “Chile Crece Contigo” program, which guarantees prenatal care, care for children during the first years of life, improvements in hospital infrastructure, and the increase of institutionalized deliveries, which currently correspond to 99% [29, 30].

In addition to the main results, we found that overall smoking prevalence decreased significantly by 19% after SBL implementation in all cities, which was steeper among younger women (20–34 years old) as has been previously reported in other studies. The relative reductions that we observed in smoking prevalence among young women (−26.5% to −31.1% in women aged 20–49 years) were greater than those recently projected by Flor et al. in the best tobacco restriction scenario (projected -12% to −15.9% in women aged 15–49 years in 175 countries), which combines the smoke-free policy with higher prices, health warnings and banned advertising [31]. Our results showed a greater change in smoking prevalence after SBL implementation in cities with higher population density (≥6,153 person/km^2^). As bigger and denser cities may present more venues for social interaction and smoking (bars, pubs, restaurants), the impact of SBL implementation in these cities could have been more extensive. Bigger/denser cities could also have more resources to enforce the SBL (more inspections, sanctions, etc.), making the SBL more impactful on health outcomes [19, 31–33]. Even though the anti-smoking law has managed to reduce the smoking prevalence in Chile, recent evidence reports low compliance in night entertainment and semi-open venues in Chile [34, 35]. The SBL has legislative gaps with no progress in a stricter proposal to tobacco restriction aligned with framework agreement of the World Health Organization, which is being discussed by the National Congress [36, 37].

We found a slight increase in the LBW rates over time at the expense of relative higher rates of late- preterm births (34–36 GW: 5.6% and 5.8% before and after SBL, respectively; data in Supplementary Table S2), which are at the higher limit compared to high income countries (3–6%) [38, 39]. This could be linked to an increase in the C-section procedures as common practice for delivery among Chilean women [40, 41]. Overall, Chile is the third country with higher cesarian section rates (∼46%) compared with the OECD countries (27.9%) [38], which is an urban phenomenon as bigger cities count with better hospital infrastructure and technical capacity. De Elejalde & Giolito reported that a reduction in delivery costs in private hospitals for women with public insurance during the last decades in Chile increased C-section probability with a negative effect on birthweight [41].

We accounted for limitations. First, the study design only allows examining short-term impacts of the SBL implementation on LBW rates. Although the null results from this study, it is important to further explore whether SBL enables conditions (such as reducing smoking prevalence among women of childbearing age) that could contribute to a reduction in the LBW rates in the long run. Further studies incorporating more detailed information on smoking prevalence and LBW rates over time could help to better understand this pathway. In addition, we suggest that future studies consider also SGA as a primary outcome of SBL implementation. Second, we were not able to account for other environmental factors influencing birthweight (such as air pollution, multidimensional indicators of poverty, or healthcare access), and other characteristics of cities influencing the enforcement of the SBL (such as density of venues where the SBL was enforced, and penalties associated with infractions to the SBL) that could help to account for between city variability in the association between SBL and LBW rates. However, we assumed that social and built environment variables did not substantially change during the study period (2011–2015) and therefore may not have significant impact in the association under study.

Finally, we were not able to retrieve more individual-level information about maternal characteristics relevant to LBW, such as individual-level smoking behavior, maternal BMI previous to pregnancy, other comorbidities during pregnancy (such as hypertension/diabetes), or previous pregnancies. Additional studies in Chile as well as in other Latin American cities need to consider these limitations when further assessing SBL interventions at the city-level.

### Conclusion

SBL implementation did not show significant impact on changes in LBW rate in Chilean cities. However, we were able to describe significant changes in smoking behavior among women of childbearing age, which is a determinant of LBW. Further studies are needed to examine the direct and indirect impact of SBL on perinatal health outcomes.
