# Supplementary material for: Smoking Ban Law in Chile: Impact in Newborns’ Birth Weight by Women’s Age Groups and by City Population Density
Source: Int J Public Health. 2022 Dec 12;67:1605087. doi: 10.3389/ijph.2022.1605087 (PMC9791390; doi:10.3389/ijph.2022.1605087)
Supplement: Supplementary file 1 [file DataSheet1.docx]

**Supplementary Figure 1. Distribution of Chilean cities by tertiles of population density (average density for the period 2011-2015, n= 21 cities)**

**
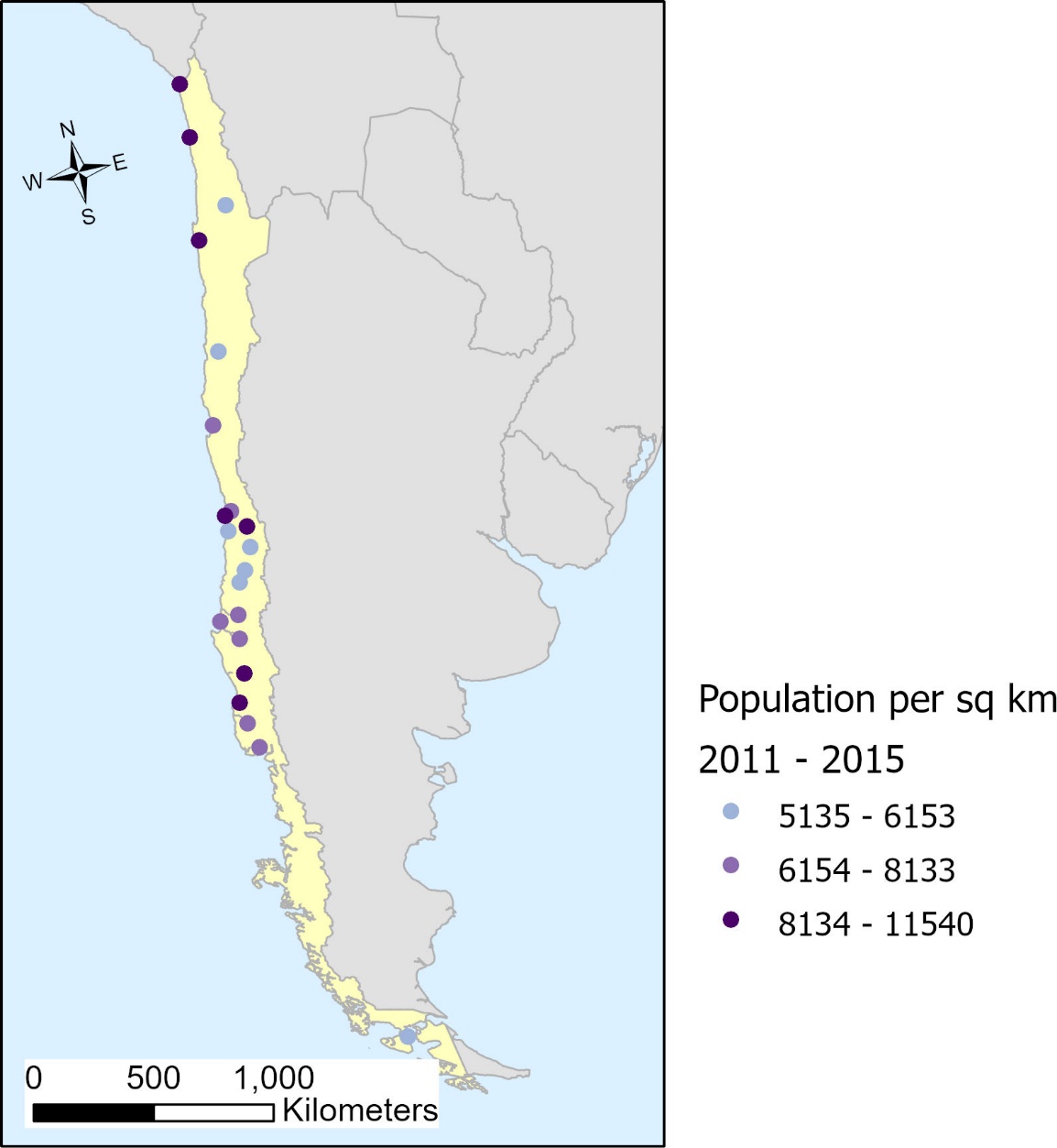
**

**Ref.** Tertile 1 included cities with a mean density ≤6153 persons/km2 (Calama, Copiapó, San Antonio, Rancagua, Talca, Curicó, Punta Arenas); Tertile 2 included cities with a mean density between 6198/8132 persons/km2 (La Serena-Coquimbo, Quillota, Concepción, Chillán, Los Angeles, Osorno, Puerto Montt), and Tertile 3 included cities with a mean density ≥8134 persons/km2 (Arica, Iquique, Antofagasta, Valparaíso-Viña del Mar, Santiago, Temuco, Valdivia)

**Supplementary Table 1. Poisson and linear mixed effect regression models assessing changes in LBW rate after the smoking ban law in all 21 cities of 100K+ residents in Chile, 2011-2015**

|  | Model 1  IRR [95% CI] | Model 2  IRR [95% CI] | Model 3  β [95% CI] | Model 4  β [95% CI] |
| --- | --- | --- | --- | --- |
| Overall |  |  |  |  |
| Intervention | 0.99 [0.93; 1.04] | 0.99 [0.93; 1.05] | -2.06 [-7.65; 3.53] | -1.35 [-5.40; 2.70] |
| Time | 1.00 [1.00; 1.01] | 1.00 [1.00; 1.01] | 0.29 [-0.12; 0.71] | 0.30 [-0.04; 0.63] |
| Intervention*Time | 1.00 [1.00; 1.01] | 1.00 [0.99; 1.01] | 0.11 [-0.36; 0.58] | 0.05 [-0.32; 0.42] |
| Stratification by city characteRISTICS |  |  |  |  |
| Tertil 1 |  |  |  |  |
| Intervention | 1.00 [0.85-1.18] | 1.00 [0.85-1.18] | -3.46 [-18.6; 11.71] | -1.52 [-15.62; 12.58] |
| Time | 1.00 [0.99-1.02] | 1.00 [0.99-1.02] | 0.05 [-0.79; 0.90] | 0.08 [-0.69; 0.85] |
| Intervention*Time | 1.00 [0.99-1.01] | 1.00 [0.99-1.01] | 0.28 [-0.93; 1.49] | 0.17 [-0.97; 1.32] |
| Tertil 2 |  |  |  |  |
| Intervention | 1.00 [0.88-1.14] | 1.00 [0.88-1.15] | 0.02 [-7.64; 7.69] | -0.25 [-7.80; 7.31] |
| Time | 1.01 [1.00-1.02] | 1.01 [1.00-1.02] | 0.43 [-0.39; 1.24] | 0.41 [-0.36; 1.18] |
| Intervention*Time | 1.01 [1.00-1.01] | 1.01 [1.00-1.01] | 0.41 [-0.02; 0.85] | -0.03 [-0.76; 0.70] |
| Tertil 3 |  |  |  |  |
| Intervention | 0.98 [0.92-1.05] | 0.98 [0.92-1.05] | -2.74 [-7.20; 1.71] | -1.94 [-7.20; 1.71] |
| Time | 1.00 [1.00-1.01] | 1.00 [1.00-1.01] | 0.39 [-0.15; 0.93] | 0.33 [-0.10; 0.75] |
| Intervention*Time | 1.00 [1.00-1.01] | 1.00 [1.00-1.01] | 0.45 [0.25; 0.64] | 0.03 [-0.34; 0.41] |
| Stratification by maternal age |  |  |  |  |
| <20 years old |  |  |  |  |
| Intervention | 0.93 [0.78-1.11] | 0.93 [0.78-1.11] | -3.90 [-21.89; 14.08] | -4.59 [-18.92; 9.74] |
| Time | 1.01 [0.99-1.02] | 1.01 [0.99-1.02] | 0.14 [-1.27; 1.56] | 0.25 [-0.94; 1.44] |
| Intervention*Time | 1.01 [1.00-1.02] | 1.01 [1.00-1.02] | 0.56 [-0.37; 1.49] | 0.62 [-0.13; 1.36] |
| 20-34 years old |  |  |  |  |
| Intervention | 0.97 [0.90-1.05] | 0.97 [0.90-1.05] | -3.71 [-10.23; 2.80] | -2.35 [-7.31; 2.60] |
| Time | 1.00 [1.00-1.01] | 1.00 [1.00-1.01] | 0.43 [-0.13; 0.99] | 0.36 [-0.07; 0.79] |
| Intervention*Time | 1.01 [1.00-1.01] | 1.00 [1.00-1.01] | 0.49 [0.09; 0.89] | 0.39 [0.09; 0.70] |
| ≥35 years old |  |  |  |  |
| Intervention | 1.09 [0.95-1.24] | 1.09 [0.96-1.24] | 5.56 [-15.02; 26.13] | 5.51 [-8.74; 19.77] |
| Time | 1.00 [0.99-1.02] | 1.00 [0.99-1.01] | -0.28 [-1.35; 0.80] | -0.03 [-0.86; 0.80] |
| Intervention*Time | 1.00 [0.99-1.01] | 1.00 [0.99-1.00] | -0.21 [-1.42; 1.02] | -0.16 [-1.01; 0.69] |

Model 1: Main approach using mixed effects Poisson regression; Model 2: Model 1 adjusted by seasonality (quarter or trimester of the year as a categorical variable); Model 3: Sensitivity analysis mixed effects linear regression analysis with LBW rate (LBW per 1000 live births) as an outcome. Model 4: Model 3 with weights for sampling size for each city (weight=sqr(number of live births)).

**Supplementary Table 2. Prevalence and relative change of preterm and small for gestational age births before and after the implementation of the smoking ban law by tertiles of city density in all 21 cities of 100K+ residents in Chile, 2011-2015.**

|  | Births before smoking ban law (January 2011-March 2013) | | | | Relative Change between before and after the implementation of SBL | | | |
| --- | --- | --- | --- | --- | --- | --- | --- | --- |
|  | **Total** | **Tertile 1** | **Tertile 2** | **Tertile 3** | **Total** | **Tertile 1** | **Tertile 2** | **Tertile 3** |
| <34 GW, n(%) | 8,943 (2.25%) | 916 (2.06%) | 1,578 (2.06%) | 6,449 (2.33%) | 3% | 2% | 11% | 1% |
| 34-36 GW, n(%) | 22,123 (5.56%) | 2,243 (5.04%) | 3951 (5.15%) | 15929 (5.75%) | 5% | 8% | 7% | 4% |
| SGA, n(%) | 38,227 (9.61%) | 4,900 (11.03%) | 6,700 (8.74%) | 26,627 (9.63%) | 1% | -1% | -1% | 1% |

SBL: Smoking Ban Law; GW: Gestational weeks at birth; SGA: Small for Gestational Age. Values expressed as absolute frequency and prevalence (%). Relative change was calculated as the difference between prevalence before and after the SBL (Relative change=post-pre)/pre).

**Supplementary Figure 2. Effect of smoking ban law implementation over low birthweight rate in all 21 Chilean cities considering a 9-month lag, 2011-2015.**


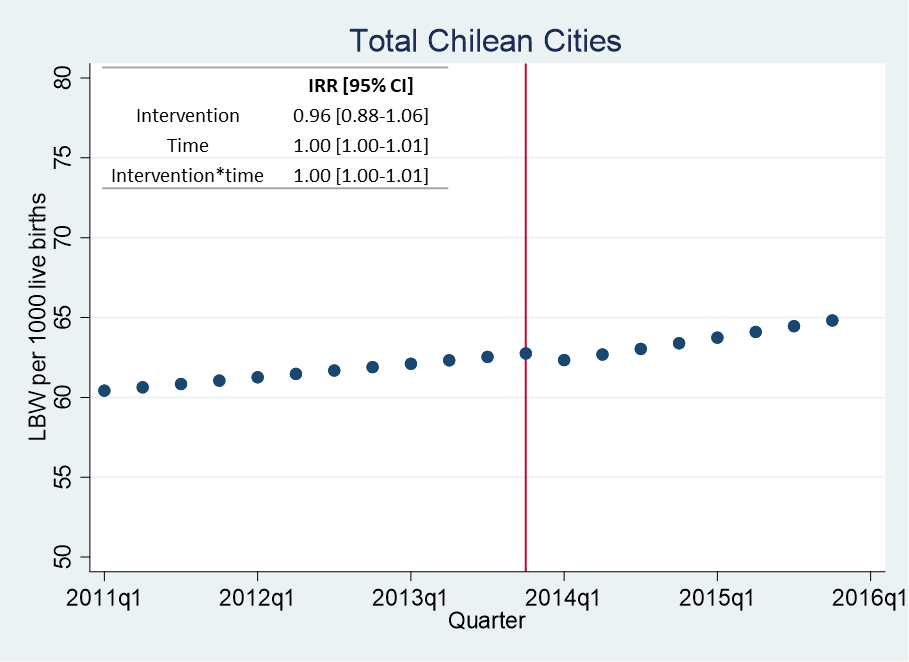


LBW: Low birth weight (birthweight <2500 grams); IRR: Incidence Rate Ratio; 95%CI: 95% Confidence Interval. Panel A shows the predicted LBW rate for all 21 Chilean cities. The red line shows the 9-month lag from the implementation of the smoking ban law (March 2013).
